# Association between tight junction proteins and cognitive performance in untreated persons with HIV

**DOI:** 10.1097/QAD.0000000000003923

**Published:** 2024-05-02

**Authors:** Francesca Bai, Valeria Bono, Lidia Borghi, Federica Bonazza, Camilla Falcinella, Virginia Vitaletti, Federica Miraglia, Mattia Trunfio, Andrea Calcagno, Jessica Cusato, Elena Vegni, Antonella d’Arminio Monforte, Giulia Marchetti

**Affiliations:** a Clinic of Infectious Diseases; b Unit of Clinical Psychology, San Paolo Hospital, ASST Santi Paolo e Carlo, Department of Health Sciences, University of Milan, Milan; c Unit of Infectious Diseases, ASST della Valle Olona, Busto Arsizio Hospital, Busto Arsizio; d Grant Office, ASST Santi Paolo e Carlo, Milan; e Clinic of Infectious Diseases; f Laboratory of Pharmacology and Pharmacotherapy, Amedeo di Savoia Hospital, Department of Medical Sciences, University of Turin, Turin, Italy.

**Keywords:** HIV infection, HIV inflammation, HIV-associated neurocognitive disorders, soluble endothelial adhesion markers, tight junction proteins, zonulin

## Abstract

**Background:**

HIV-associated neurocognitive disorders (HAND) still affects persons with HIV (PWH) and their pathogenesis is not completely understood. We aimed to explore the association between plasma and cerebrospinal fluid (CSF) markers of blood–brain barrier (BBB) impairment and HAND in untreated PWH.

**Design:**

Cross-sectional study.

**Methods:**

We enrolled untreated PWH, who underwent blood examinations and lumbar puncture to measure inflammation (IL-15, TNF-α), BBB damage (zonulin and tight junction proteins, tight junction proteins: occludin, claudin-5) and endothelial adhesion molecules (VCAM-1, ICAM-1). A comprehensive neurocognitive battery was used to diagnose HAND (Frascati criteria).

**Results:**

Twenty-one patients (21/78, 26.9%) patients presented HAND (100% ANI). HAND patients displayed more frequently non-CNS AIDS-defining conditions, lower nadir CD4^+^ T cells and increased CD4^+^ T-cell exhaustion (lower CD4^+^CD127^+^ and CD4^+^CD45RA^+^ T-cell percentages), in comparison to individuals without cognitive impairment. Furthermore, HAND was characterized by higher plasma inflammation (IL-15) but lower CSF levels of biomarkers of BBB impairment (zonulin and occludin). The association between BBB damage with HAND was confirmed by fitting a multivariable logistic regression. CSF/plasma endothelial adhesion molecules were not associated with HAND but with a poor performance in different cognitive domains.

**Conclusion:**

By showing heightened inflammation and BBB impairment, our study suggests loss of BBB integrity as a possible factor contributing to the development of HAND in untreated PWH.

## Introduction

HIV-associated neurocognitive disorders (HAND) still represent a rather common complication of HIV infection. Despite being more frequently observed in late presenters with low CD4^+^ T-cell counts at diagnosis, HAND persists during virally suppressive combination antiretroviral therapy (cART). A recent meta-analysis found that the overall prevalence of HAND was 42.6% [95% confidence interval (CI) 39.7–45.5%)], with a predominance of asymptomatic neurocognitive impairment (ANI, 23.5%, 95% CI 20.3–26.8%), according to the Frascati criteria [1–4]. New approaches for the diagnosis and classification of cognitive impairment in persons with HIV (PWH) are under investigation, as Frascati criteria could lead to an overestimation, but a new standardized definition has not yet be identified [5]. The most important risk factors for HAND are low CD4^+^ T-cell nadir and AIDS-defining diseases [6]. Although patients diagnosed with ANI are asymptomatic by definition, they show a two-fold to six-fold greater risk of evolution to symptomatic and more severe forms, compared with patients without cognitive impairment, underlining the need to follow up these patients [7,8].

From a pathogenetic standpoint, HIV enters the central nervous system (CNS) from the earliest stages of infection because of increased permeability of blood brain barrier (BBB) [9,10]. A decline in adaptive immunity with depletion of CD4^+^ T-cell counts and an active HIV replication leads to peripheral inflammation; thus, viral proteins, microbial translocation products (LPS/sCD14), and pro-inflammatory cytokines, such as HIV-induced IL-6, IL-10, TNF-α, and IL-15, are able to increase BBB permeability through increased expression of adhesion molecules and destruction of tight junction proteins [11,12]. Tight junction proteins are a group of different proteins that provides a physical barrier forming a belt-like structure between adjacent endothelial cells in gut–blood barrier and BBB; they involve occludin, claudin (claudin-1, claudin-3, claudin-5, and claudin-12), the membrane-associated guanylate kinase tight junction protein zonula occludens – ZO1, ZO2, and ZO3, and the junctional adhesion molecules [13–15]. Perturbations in tight junction protein expression are well described in course of HIV infection, with a decrease in the tight junction protein expression in brain and gut biopsies of PWH [12–14,16]. The soluble protein zonulin plays a key role in regulating the gut–brain axis and the functioning of tight junction proteins, separating ZO proteins from the other tight junction proteins such as occludin and thus increasing permeability. An increased intestinal release of zonulin in the systemic circulation is associated with impaired gut permeability and systemic inflammation; zonulin and inflammation mediators reach the CNS leading to increased BBB permeability and neuroinflammation [17]. In fact, an increased intestinal zonulin release has been described in the course of several gastrointestinal diseases and HIV infection and it is associated with biomarkers of gut–blood barrier and BBB damage [17,18]. BBB-increased permeability allows the entry of virus, activated lymphocytes, monocytes, and neurotoxic cytokines in the CNS and finally causes neuroinflammation, progressive neurological damage, and cognitive impairment [19]. The establishment of a viral reservoir in the CNS, characterized by a higher viral load in CSF compared with plasma viremia (CSF to plasma HIV-RNA ratio >1), has been previously found as a risk factor for HAND [20].

In this context, our hypothesis was that increased HIV-driven inflammation in both the periphery and the CNS, together with an impaired BBB permeability due to higher expression of endothelial adhesion molecules and reduced expression of zonulin and tight junction proteins, is associated with cognitive deficits in untreated PWH. We thereby aimed to study the association between HAND and peripheral and CSF levels of pro-inflammatory cytokines (IL-15, TNF-α), endothelial adhesion molecules (VCAM-1, ICAM-1), zonulin and tight junction proteins (occludin, claudin-5).

## Materials and methods

### Study population

We conducted a cross-sectional study at San Paolo Hospital, ASST Santi Paolo e Carlo, Department of Health Sciences, University of Milan, Italy. Patients with a new or recent diagnosis of HIV infection and before combination antiretroviral therapy (cART) introduction were consecutively enrolled at our center from January 2016 to December 2019. The inclusion criteria were: age older than 18 years, being naive to antiretroviral treatment, any CD4^+^ count and plasma/CSF HIV-RNA, comprehension and speaking of Italian language, and signature of written informed consent. Patients were excluded from the study if they presented cirrhosis, alcohol or other substance abuse, neurological or psychiatric diseases, or ongoing or previous CNS opportunistic infections.

All enrolled patients underwent a complete neurocognitive evaluation performed by a trained neuropsychologist, blood examinations, and lumbar puncture. Demographic, clinical, and other viro-immunological parameters, were collected from the clinical records. The study was approved by the Ethics Committee Area 1 Milan (n 476, 06/04/2018).

### Neuropsychological assessment

The comprehensive neurocognitive evaluation included 11 tests, exploring six main cognitive domains. The following tests were used: speed of information processing (Trail Making Test Part A, Symbol Digit Modality Test, Stroop Color Test – Time); learning and memory (Rey Auditory Verbal Learning Test Immediate Recall and Delayed Recall, Rey–Osterrieth complex Delayed Recall); abstraction/executive functioning (Stroop Color Test – Error, Trail Making Test Part B, Rey–Osterrieth complex Figure Copy); verbal fluency (Semantic and Phonemic Fluency); attention/working memory (Corsi's block Tapping Test, Forward and Backward Digit span, Trail Making Test Part BA); and motor skills (Finger Tapping Test). Raw scores on each test were calculated. Scores were corrected for age, educational level, and gender using Italian normative data. The autonomy in the activities of daily living was assessed by Instrumental Activities of Daily Living Scale (IADL). On the basis of the scores of these tests, patients were diagnosed with HAND, as defined by 2007 Frascati criteria [1]. HAND spectrum included ANI, mild neurocognitive disorders (MND), and HIV-associated dementia (HAD).

The raw scores were then converted to normative *T* scores. *T* scores were calculated for each test [mean = 50 and SD = 10; *T* = 50 + 10(*x* − mean/SD)] and for cognitive domains. Domain-specific *T* scores were calculated averaging the *T* scores of all the tests included in the domain. Patients were also asked to complete Hospital Anxiety and Depression Scale (HADS) [21] investigating the symptoms of anxiety and depression and 36-Item Short Form Health Survey (SF-36) for Health-related Quality of Life (HrQoL) [22].

HADS consists of two subscales, Anxiety and Depression, and includes 14 items on a 4-point Likert scale (range 0–3), seven items for the anxiety subscale (HADS Anxiety), and seven for the depression subscale (HADS Depression); a score higher than 8 points in HADS identified anxiety (HADS-A) or depression (HADS-D) symptoms. SF-36 is a questionnaire based on eight multiitem health domains (35 items): physical functioning (10 items), bodily pain (2 items), limitations due to physical health problems and personal or emotional problems (7 items), bodily pain (2 items), emotional well being (5 items), social functioning (2 items), fatigue (4 items), and general health perceptions (5 items). These eight scales are then aggregated into two summary measures: Mental Health Index (MHI) and Physical Health Index (PHI). The 36th item that provides an indication of perceived change in health status, is not included in the summary scores. The responses are scored in a two-step process: first, precoded numeric values are recoded per the scoring validated key and each item is scored on a 0–100 range; then, in step 2, items in the same scale are averaged together to create the eight scale scores. Higher scores define a more favorable health status. There is not a standardized cut-off, but a database of normative data is available to calculate the norm-based score that identifies the impaired cases.

### Blood examinations and lumbar puncture

All participants underwent blood examinations to detect plasma HIV-RNA and CD4^+^/CD8^+^ T-cell count and lumbar puncture to quantify CSF HIV-RNA by the ultrasensitive Abbott RealTime HIV-1 assay (Abbott Laboratories, Des Plaines, Illinois, USA) on frozen samples (lower detection limit of <40 copies/ml). CSF to plasma HIV-RNA ratio was calculated by dividing CSF HIV-RNA by plasma HIV-RNA.

### Peripheral and cerebrospinal fluid inflammation, endothelial adhesion molecules, and blood–brain barrier impairment

Peripheral blood samples were collected in EDTA tubes from all study participants; plasma was separated by centrifugation and stored at −80 °C to perform the subsequent analysis.

We measured the following soluble biomarkers in blood and CSF samples: inflammatory cytokines (IL-15, TNF-α), endothelial adhesion molecules (VCAM-1, ICAM-1), zonulin and tight junction proteins (claudin-5 and occludin).

Plasma and CSF levels of TNF-α, zonulin, and tight junction proteins (occludin, claudin-5) were quantified by commercially available ELISA assay (TNF-α: R&D Systems, Minneapolis, Minnesota, USA; zonulin, occludin, and claudin-5: MyBioSource, San Diego, California, USA), according to the manufacturer's protocol. The ELISA detection range was: Claudin – 5 2–0.0625 ng/ml; Occludin 10–0.156 ng/ml; Zonulin 800–1 ng/ml, and TNF-α 10–0.2 pg/ml.

Plasma and CSF levels of IL-15, ICAM-1, and VCAM-1 were measured by LUMINEX technology (R&D Systems), according to manufacturer's instructions.

### T-cell immune phenotypes

T-lymphocyte surface phenotypes were evaluated on cryo-preserved samples by flow cytometry. 1.5 × 10^6^ of thawed PBMCs were plated in complete Roswell Park Memorial Institute (RPMI) medium containing 10% human serum supplemented with 1% Penicillin–Streptomycin–Glutamin. Overnight-rested PBMCs were stained with the appropriate antibodies for 20 min at 4 °C in the dark and acquired using FACSVersecytometer (Becton Dickinson Italia Spa, Milan, Italy). The following fluorochrome-labeled antibodies were used: CD4 PE-Cy7, CD8 PE-Cy5, CD38 PE, CD45R0 APC, CD45RA FITC and CD127 PE (BD Biosciences). The following combination of antibodies was used: CD8/CD4/CD127/CD45RA (maturation), CD8/CD4/CD38/CD45R0 (activation). Cell viability was assessed by 7-aminoactynomycin D (7-AAD; BD Biosciences): only samples with a viability greater than 70% were used for the experiments.

### Statistical analyses

Categorical variables were presented as absolute numbers (percentages), quantitative variables as medians (interquartile range, IQR). Comparison of plasma and CSF biomarkers between patients with HAND and individuals without cognitive deficits was performed by Mann–Whitney and chi-square or Fisher's exact test, as appropriate. The significant association between peripheral inflammation or biomarkers of BBB alteration and HAND was investigated by fitting multivariable logistic regression models, adjusting for possible confounders (non-CNS AIDS-defining diseases, nadir CD4^+^ T-cell counts, CSF/plasma HIV-RNA ratio and age). The association between plasma and CSF biomarkers and domain-specific *T* scores was explored by univariable and multivariable linear regression, adjusting for the same confounders. Statistical analyses were performed by STATA software, version 14.0.

## Results

### Demographic and clinical factors associated with HIV associated neurocognitive disorders

Seventy-eight PWH at HIV diagnosis and before cART introduction were consecutively enrolled. Baseline characteristics of study population, according to HAND diagnosis, are shown in Table 1; 21/78 (26.9%) were diagnosed with HAND according to Frascati criteria [1]. All HAND patients (21/21, 100%) were affected by ANI. The results of the neurocognitive evaluation with raw scores and *T* scores for each test and for cognitive domains are shown in the Supplementary Table 1.

**Table 1:** Demographic, viro-immunological, and neuropsychological characteristics of the study population according to HIV-associated neurocognitive disorders.

	Study population (*N =* 78)	No HAND (*N* = 57)	HAND (*N =* 21)	*P* values
Demographic parameters				
Age (years)^a^	45 (37–54)	44 (36–52)	47 (39–56.5)	0.191
Sex (male individuals)^b^	71 (91%)	52 (91.2%)	19 (90.5%)	0.918
Days from HIV diagnosis to study enrolment^a^	69 (19–630)	76 (31–546)	32 (9–709)	0.199
Risk factor for HIV^b^				0.871
MSM	56 (71.8%)	40 (70.2%)	16 (76.2%)	
Heterosexual contacts	13 (16.7%)	10 (17.5%)	3 (14.3%)	
Ex IDUs	9 (11.5%)	7 (12.3%)	2 (9.5%)	
HCV Ab positive^b^	5 (6.4%)	2 (3.5%)	3 (14.3%)	0.122
HBsAg positive^b^	1 (1.3%)	1 (1.8%)	0	1.000
AIDS-defining conditions^b^	17 (21.8%)	8 (14%)	9 (42.9%)	**0.007**
Comorbidities^b^	27 (34.6%)	20 (35.1%)	7 (33.3%)	0.845
CSF parameters				
WBC (cells/μl)^a^	0 (0–8)	0 (0–7)	5 (0–12)	0.153
Protein count (mg/dl)^a^	52.2 (37–69.25)	48 (37–59)	65 (38.75–84.25)	0.111
Viro-immunological parameters				
CD4^+^ T-cell nadir (cells/μl)^a^	297 (123–470)	333 (236–492)	106 (28–440)	**0.026**
CD4^+^ T cells (cells/μl)^a^	394 (226–526)	333 (233–504)	116 (40–455)	**0.021**
CD4^+^/CD8^+^ ratio^a^	0.36 (0.14–0.61)	0.36 (0.14–0.72)	0.25 (0.14–0.52)	0.494
Log_10_ HIV-RNA copies/ml plasma^a^	4.34 (3.78–4.89)	4.35 (3.7–4.87)	4.31 (4.05–5.59)	0.359
Log_10_ HIV-RNA copies/ml CSF^a^	3.68 (2.89–4.17)	3.64 (2.8–4.08)	3.88 (2.9–4.95)	0.276
CSF/plasma HIV-RNA ratio^a^	0.8 (0.65–0.97)	0.79 (0.66–0.95)	0.86 (0.58–1.08)	0.605
CSF/plasma HIV-RNA ratio^b^				0.45
<1	53/66 (80.3%)	43/52 (82.7%)	10/14 (71.4%)	
≥1	13/66 (19.7%)	9/52 (17.3%)	4/14 (28.6%)	
Neuropsychological parameters				
SF-36 MHI^b^	25/61 (41%)	21/48 (43.8%)	4/13 (30.8%)	0.399
SF-36 PHI^b^	7/61 (11.5%)	3/48 (6.3%)	4/13 (30.8%)	**0.014**
HADS-D^b^	1 (1.3%)	1 (1.8%)	0	1.000
HADS-A^b^	7 (9%)	6 (10.5%)	1 (4.8%)	0.667

A, anxiety; CSF, cerebrospinal fluid; D, depression; ex IDUs, ex intravenous drug users; HADS, Hospital Anxiety and Depression Scale; HAND, HIV-associated neurocognitive disorders; MSM, men who have sex with men; SF-36 MHI, Mental Health Impairment; SF-36 PHI, Physical Health Impairment (SF-36 questionnaire); WBC, white blood cells.

a Quantitative parameters are presented as median, interquartile range (IQR); Mann–Whitney test for comparison between patients diagnosed with HAND and patients with normal neurocognitive evaluation.

b Qualitative parameters are presented as absolute numbers, percentages; chi-square test or exact Fisher's test for comparison, as appropriate. Bold values indicate statistical significance.

As expected, HAND patients were more likely to present non-CNS AIDS-defining diseases (Table 1); AIDS conditions were: *Pneumocystis jirovecii* pneumonia (7/17, 41%), Kaposi sarcoma (2/17, 12%), esophageal candidiasis (2/17, 12%), *Mycobacterium avium* complex (MAC) disease (1/17, 6%), disseminated shingles (1/17, 6%), and wasting syndrome (4/17, 23%). As regards neuropsychological outcomes, no differences in symptoms of anxiety and depression by HADS were observed between the two groups; patients with HAND more frequently showed altered HrQoL scores by SF-36, specifically PHI (Table 1).

### Peripheral CD4^+^ and CD8^+^ T-cell immune phenotypes according to HIV associated neurocognitive disorders

**Fig. 1:**
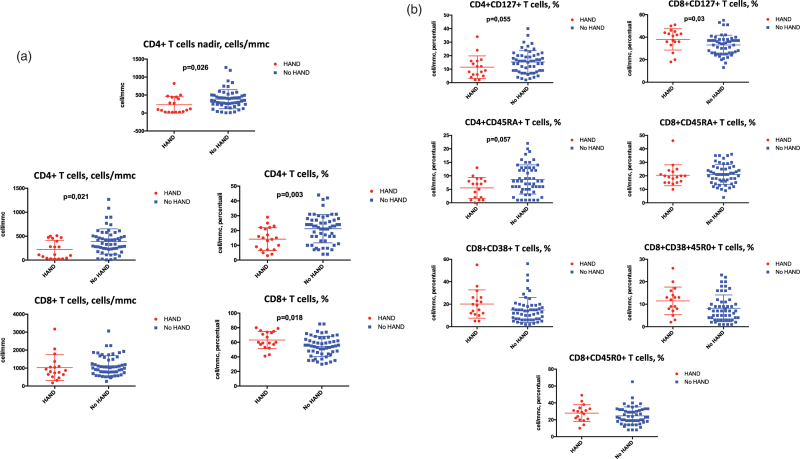
Peripheral T-cell phenotypes according to HIV-associated neurocognitive disorders.

Compared with individuals without HAND, patients diagnosed with HAND also presented lower current and nadir CD4^+^ T-cell counts and higher CD8^+^ T-cell percentages (Fig. 1a). Peripheral CD4^+^ and CD8^+^ T-cell subsets were impaired in HAND, with a reduction of central memory CD4^+^ T-cell percentages expressing IL-7 receptor (CD4^+^CD127^+^%) and of naïve CD4^+^ T cells (CD4^+^CD45RA^+^%), whereas CD8^+^CD127^+^ percentages were increased (Fig. 1b). No differences in activated and terminally differentiated CD4^+^ and CD8^+^ T-cell immune phenotypes were reported between HAND individuals and patients with a normal cognitive function (Fig. 1).

Peripheral CD4^+^ and CD8^+^ T cells (a) and T-cell subsets (b) in patients with HAND (red color) and patients without cognitive disorders (blue color). Mann–Whitney test for comparison between the two groups of patients; *P* values less than 0.05 are shown. Each square indicated the value of a single patient, median and interquartile range are also illustrated. CD4^+^ T-cell nadir, CD4^+^ T-cells and percentages, CD8^+^ T cells and percentages. Percentages of CD4^+^CD127^+^, CD8^+^CD127^+^, CD4^+^CD45RA^+^, CD8^+^CD45RA^+^, CD8^+^CD38^+^, CD8^+^CD38^+^45R0^+^, CD8^+^CD45R0.^+^ T cells. HAND, HIV-associated neurocognitive disorders

### Peripheral and cerebrospinal fluid biomarkers of inflammation, blood–brain barrier integrity, and endothelial adhesion

**Fig. 2:**
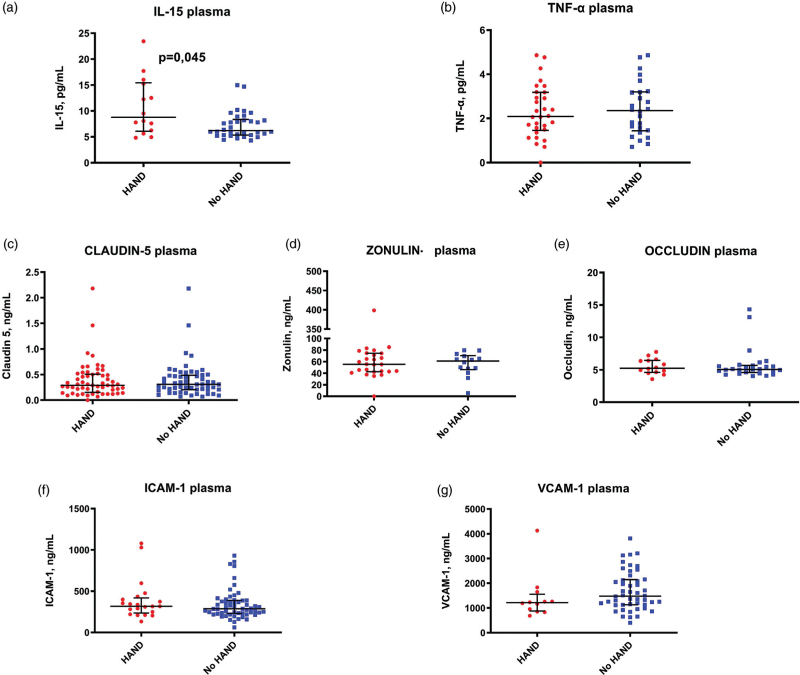
Plasmatic levels of pro-inflammatory cytokines, endothelial adhesion molecules, zonulin, and tights junction proteins according to HIV-associated neurocognitive disorders.

**Fig. 3:**
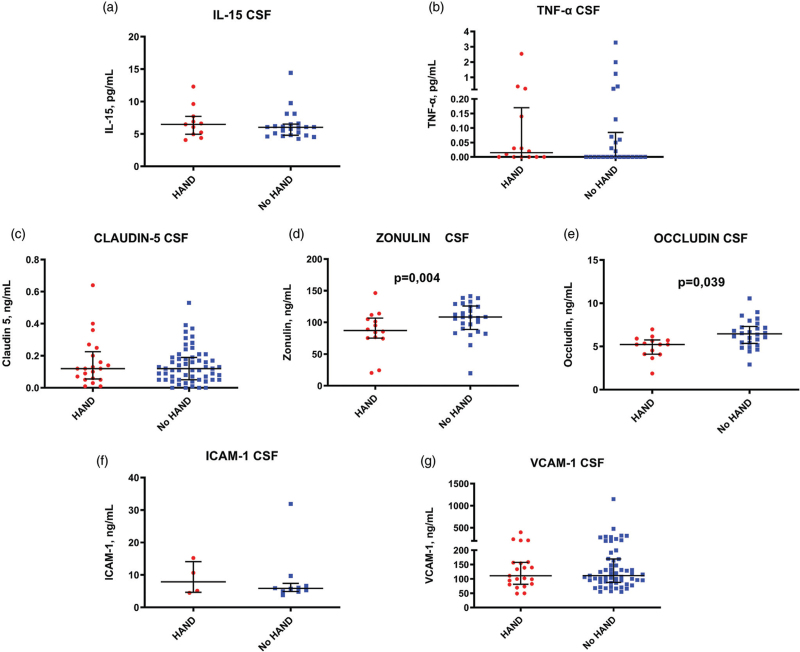
Cerebrospinal fluid levels of pro-inflammatory cytokines, endothelial adhesion molecules, zonulin, and tight junction proteins according to HIV-associated neurocognitive disorders.

We next explored peripheral and CSF inflammation by measuring circulating levels of IL-15 and TNF-α; higher plasma levels of IL-15 were observed in patients with HAND, whereas CSF IL-15 and plasma and CSF levels of TNF-α were similar between the two groups (Figs. 2 and 3, panels a and b). Interestingly, although no differences were shown in circulating zonulin and tight junction proteins (Fig.2, panels c–e), concentrations of zonulin and occludin in CSF were significantly lower in patients with HAND as compared with individuals without cognitive impairment (Fig. 3, panel c–e). Finally, we did not observe any difference in plasma and CSF levels of soluble endothelial adhesion molecules (Figs. 2 and 3, panel f and g). Supplementary Figure 1 shows the different patterns of inflammation and BBB impairment biomarkers according to HAND.

Peripheral levels of inflammatory cytokines (IL-15, TNF-α; panels a and b), zonulin and tight junction proteins (claudin 5, occludin; panels c–e), and endothelial adhesion molecules (VCAM-1, ICAM-1; panels f and g) in patients with HAND (red color) and patients without cognitive disorders (blue color). Mann–Whitney test for comparison between the two groups of patients; *P* values less than 0.05 are shown. Each square indicated the value of a single patient, median and interquartile range are also illustrated. (a) Plasma levels of IL-15; (b) plasma levels of TNF-α; (c) plasma levels of claudin-5; (d) plasma levels of zonulin; (e) plasma levels of occludin; f) plasma levels of ICAM-1; and (g) plasma levels of VCAM-1. HAND, HIV-associated neurocognitive disorders.

CSF levels of inflammation biomarkers (IL-15, TNF-α panels a and b), tight junction proteins (claudin-5, occludin, and zonulin; panels c–e), and cell adhesion molecules (VCAM-1, ICAM-1) (panels f and g) in patients with HAND (red color) and patients without cognitive disorders (blue color). Mann–Whitney test for comparison between the two groups of patients; *P* values less than 0.05 are shown. Each square indicated the value of a single patient, median and interquartile range are also illustrated. (a) CSF levels of IL-15; (b) CSF levels of TNF-α; (c) CSF levels of claudin-5; (d) CSF levels of zonulin; (e) CSF levels of occludin; (f) CSF levels of ICAM-1; and (g) CSF levels of VCAM-1. CSF, cerebrospinal fluid; HAND, HIV-associated neurocognitive disorders.

### Peripheral inflammation and impaired permeability of blood–brain barrier are associated with HIV associated neurocognitive disorders

In order to better understand the association between inflammation, BBB impairment and HAND, we performed multivariable logistic regression analyses: increased IL-15 plasma levels were associated with HAND, but without reaching statistical significance after adjustment for possible confounders (Table 2a). A weak association between lower CSF zonulin and occludin levels with HAND was confirmed after adjustment for age, non-CNS AIDS events, CD4^+^ nadir and CSF/plasma HIV-RNA ratio (Table 2b and c).

**Table 2:** Association between peripheral inflammation, blood–brain barrier impairment, and HIV-associated neurocognitive disorders by fitting a multivariable logistic regression analysis.

(a) Parameters, model 1	Adjusted odds ratio	95% CI	*P* values
Age, each year more	0.998	0.994–1.002	0.235
AIDS-defining condition			
Yes	1		0.207
No	0.331	0.059–1.846	
CD4^+^ T-cell nadir (each cell/mmc more)	0.998	0.994–1.002	0.235
CSF/plasma HIV-RNA ratio			
≥1	1		0.704
<1	0.736	0.151–3.581	
Plasma IL-15 (each log_10_ pg/ml more)	1.142	0.976–1.337	0.097

(b) Parameters, model 2	Adjusted odds ratio	95% CI	*P* values
Age, each year more	0.934	0.782–1.116	0.452
AIDS-defining condition			
Yes	1		0.283
No	0.069	0.001–9.071	
CD4^+^ T-cell nadir (each cell/μl more)	0.978	0.957–1.000	0.046
CSF/plasma HIV-RNA ratio			
≥1	1		0.633
<1	0.398	0.009–17.428	
CSF zonulin (each log_10_ ng/ml more)	0.910	0.830–0.997	0.043

(c) Parameters, model 3	Adjusted odds ratio	95% CI	*P* values
Age, each year more	1.113	0.912–1.357	0.292
AIDS-defining condition			
Yes	1		0.168
No	0.045	0.001–3.685	
CD4^+^ T-cells nadir (each cell/mmc more)	0.994	0.985–1.004	0.271
CSF/plasma HIV-RNA ratio			
≥1	1		0.124
<1	0.036	0.001–2.498	
CSF occludin (each ng/ml more)	0.234	0.062–0.885	0.032

Multivariable logistic regression analysis; (a) model 1: association between plasma IL-15 and HAND; (b) model 2: association between CSF zonulin and HAND; (c) model 3: association between CSF occludin and HAND. All the three models are adjusted for age, CD4^+^ T-cell nadir, CSF/plasma HIV-RNA ratio, and AIDS diseases. CI, confidence interval; CSF cerebrospinal fluid.

### Inflammation and blood–brain barrier impairment are associated with cognitive performance

We then also explored the association between the different biomarkers of inflammation and BBB impairment with cognitive performance on the main cognitive domains. Supplementary Table 2, shows the association between plasma and CSF levels of the biomarkers and the domain-specific *T* scores. We found some associations between inflammation, endothelial adhesion molecules, and tight junction proteins with poor performance in several cognitive domains in the univariable linear regression analysis; by fitting a multivariable analysis, adjusting for possible confounders (age, CD4^+^ T-cell nadir, CSF/plasma HIV-RNA ratio, and AIDS diseases), we confirmed that CSF IL-15 was associated with poor performances on attention, plasma/CSF endothelial adhesion molecules were associated with poor performances on processing speed, and motor skills and plasma claudin-5 was associated with poor performance on processing speed (Supplementary Table 2).

## Discussion

In our study, we found that: 26.9% untreated PWH of our cohort were diagnosed with HAND; all of them were found at the asymptomatic stage (ANI) but already experienced an impact on HrQoL.

HAND was more common in patients with a late diagnosis of HIV infection, characterized by the presence of non-CNS AIDS-defining illnesses, low nadir and current CD4^+^ T cell counts and reduced naive and central memory compartments.

Peripheral inflammation, demonstrated by higher circulating levels of pro-inflammatory cytokines (IL-15), and decreased CSF concentrations of soluble occludin and zonulin seems to characterize antiretroviral-naive patients with HAND.

Neuroinflammation and BBB impairment (increased plasmatic levels of claudin-5, CSF levels of IL-15, and CSF/plasma levels of endothelial adhesion molecules) seems associated with poor cognitive performances.

Although the incidence of HAND and, specifically, of the more severe forms of dementia, has significantly decreased in recent years, still 20–30% of PWH displays cognitive deficits worldwide, rising up to 50% when focusing on patients with AIDS-defining conditions [23]. According to the literature, most patients currently display ANI [2]. In our study, we confirmed that nearly one-third of newly diagnosed patients display some degree of cognitive impairment before cART introduction; more specifically, all subjects of our study were diagnosed with ANI by Frascati criteria [24]. However, we confirm that even the asymptomatic disorders have an impact on quality of life, as demonstrated by a reduced physical health index of the SF-36 questionnaire, which is in line with data reported by previous studies [25]. No specific treatment is indicated for this condition other than antiretroviral treatment, even though it is widely recognized that these mild disorders might evolve into symptomatic and more severe impairment over the years [7,8,26].

Well known risk factors for cognitive disorders are opportunistic infections and low CD4^+^ T-cell counts; indeed, a late presentation of HIV infection can be associated with severe cognitive impairment. Still today, this represents a major clinical problem in Europe, as up to 50.4% of patients are diagnosed with AIDS or with CD4^+^ T-cell count below 350 cells/μl [27]; similar figures are recorded in Italy (57.4–66.2) [28].

We next sought to investigate the immune and pro-inflammatory milieu in both the periphery and the CSF. Interestingly, peripheral T-lymphocyte immune phenotyping revealed a significant contraction of the naive and central memory CD4^+^ T-cell subset in HAND patients. Indeed, a reduced production and increased depletion of naive and central memory CD4^+^ T cells seems to characterize advanced presentation of HIV infection and cognitive impairment. The persistence of long-term defects of T-cell function, even after starting virally effective cART, together with the maintenance of a suboptimal control of the infection and of the viral reservoirs, still represents an obstacle to the effective cure of HAND and, more generally, of HIV infection itself [29].

Interestingly, we found that higher levels of IL-15 in periphery are associated with HAND, though this association was finally lost after correction for possible confounders; similarly, neuroinflammation was associated with cognitive deficits in attention and working memory. Several studies have reported an association between peripheral and central inflammation, measured by different cytokines or monocyte activation markers, and cognitive decay in HIV infection [30–32]. IL-15 plays an important role in HIV infection, has been associated with viremia, and other markers of immune activation, specifically activating and expanding CD8^+^ T cells [33,34], and some previous data have shown an association between this cytokine and neurological impairment in cART-treated PWH, mainly with associated vascular disorders [35].

Previous studies focusing on other CNS diseases have found a similar association between IL-15 and cognitive deficits: for instance, in acute lymphoblastic leukemia a peripheral increase in pro-inflammatory cytokines, including IL-15, was associated with increased expression of adhesion molecules by BBB endothelial cells, which leads to entry of neoplastic cells and activated lymphocytes into CNS, neuroinflammation, and eventually cognitive damage [36,37]. Heightened plasma and CSF IL-15 has also been described in patients suffering from multiple sclerosis: peripheral inflammation would facilitate the expression of chemokines and adhesion molecules on CD4^+^ T cells, thus allowing their migration into CNS [38]. In HIV infection, the secretion of cytokines by BBB pericytes creates a pro-inflammatory environment that may potentiate the ability of the virus to cross the BBB [11].

An increased expression of endothelial adhesion molecules (ICAM-1 and VCAM-1) in BBB was observed during the autopsies of persons with HIV (PWH) with encephalitis; similarly, in mouse models, brain endothelial cells expressed high concentrations of ICAM-1 and VCAM-1 after contact with viral proteins, such as gp120 [39–41]. Serum and CSF levels of soluble intercellular adhesion molecules were also studied in the early years of HIV pandemic: serum levels were increased in PWH compared with HIV-negative ones, reaching the highest concentrations in patients affected by HIV encephalopathy. CSF concentrations of soluble ICAM-1 were increased only in PWH with neurological diseases, probably because of a passive flow from blood through an impaired BBB [42]; similarly, CSF-soluble forms of adhesion molecules also increased in course of sepsis, CNS inflammation, such as meningitis and dementia [43]. We did not observe a direct association between endothelial adhesion molecules and HAND probably because of the small sample size of our study population, but a negative correlation between plasma and CSF levels of VCAM-1 and ICAM-1 with poor cognitive performances have been shown also in our cohort.

BBB disruption characterizes several neurological diseases, including CNS viral infections and HIV itself [15,44–46]. Protein–protein interactions of the tight junction proteins represent one of the main mechanisms regulating BBB permeability; endothelial cells of cerebral microvessels use tight junction proteins to protect CNS from harmful substances circulating in the blood and maintain brain homeostasis [47]. Two of the main components of tight junction proteins in BBB are claudin-5 and occludin [36]. HIV infection was associated with increased circulating levels of IL-6, which in turn could reduce the expression of tight junction proteins in BBB; a fragmentation of tight junction proteins, in association with astrocytosis and activated monocytes, has been reported in PWH suffering from encephalitis. Serum and CSF levels of soluble tight junction proteins and their serum/CSF ratio correlated with CNS involvement in patients diagnosed with leukemia [36], whereas higher CSF concentrations were reported after brain injury following intracranial hemorrhage or ischemic insults [48,49]. Finally, preliminary studies have shown a correlation between BBB alterations and cognitive impairment [50]. No previous data about the association between peripheral or CSF levels of soluble tight junction proteins and HAND in PWH are available.

Zonulin is a protein that modulates tight junctions’ molecular assembly and regulates the integrity and functioning of intestinal–epithelial and BBB [17,51]. It has been studied as marker of gut–epithelial barrier integrity and enterocyte function and is involved in paracellular intestinal permeability [52]. Increased plasmatic levels of zonulin have not only been observed in PWH but also in different immune diseases, chronic gut inflammation, and dementia [51,53–56]. Furthermore, heightened concentrations of circulating zonulin are strong predictors of mortality in cART-treated PWH [57,58].

Plasmatic and CSF levels of zonulin and tight junction proteins may reflect loss of gut and BBB integrity and could be useful biomarkers of impaired gut–brain axis and HAND. In our study, we describe lower zonulin and occludin levels in the CSF of patients with HAND, to possibly reflect a disrupted gut–brain axis and BBB damage. Since the first hours after HIV infection, some viral proteins such as Tat and gp120 could reduce the expression of tight junction proteins in the BBB and the loss of these proteins could explain the reduction of CSF levels that we observed [15,59]. We also observed a positive association between plasmatic levels of claudin-5 and poor performances on some cognitive domains; the exposure of the BBB to circulating zonulin is known to increase BBB permeability, thanks to the activation of zonulin receptors and the separation of occludin from zonula occludens proteins. This disruption of BBB integrity has been already associated with increased circulating levels of claudin-5 in patients with neurodegenerative or neuroinflammatory diseases [17,18]. Future studies about a possible impact of virally effective cART in reducing inflammation and immune activation both in periphery and in the CNS and in restoring BBB impairment and gut–brain axis with a subsequent cognitive improvement are crucial.

Our study has some limitations: the small sample size, especially for some biomarkers; possible unmeasured confounders in the multivariable analysis; the absence of other biomarkers of BBB impairment, such as CSF/serum albumin ratio, CSF/protein count or sCD163; the absence of mild/severe forms of HAND in our cohort and the use of HAND criteria that might overestimate the cognitive impairment in PWH; the lack of more specific gut barrier and gut–brain axis investigations that would broaden our understanding of gut and brain barrier permeability and of their reciprocal interactions in the pathogenesis of HIV-driven neurocognitive impairment; and the lack of a control group of persons without HIV.

In conclusion, our study reveals that patients diagnosed with HAND feature a depleted naïve and central memory CD4^+^ T-cell compartment, peripheral inflammation, and BBB damage. Increased BBB permeability is associated with reduced CSF levels of zonulin and occludin and increased plasmatic levels of claudin-5. Further studies are needed to understand whether in patients with HAND BBB impairment, measured by plasma and CSF levels of zonulin and tight junction proteins, is partially restored by introduction of cART or to investigate new therapeutic options targeting tight junction proteins and gut–brain axis. Indeed, given data from large cohorts demonstrating an association between altered circulating levels of zonulin and mortality in cART-treated PWH [57,60], a fine understanding of the possible clinical exploitation of plasma/CSF zonulin and tight junction protein measurements as biomarkers of HAND is needed.

## Acknowledgements

We are thankful to all the patients who participated in the study and their families. We would like to thank all the staff of the Clinic of Infectious Diseases and Tropical Medicine, San Paolo Hospital, ASST Santi Paolo e Carlo, Department of Health Sciences, University of Milan who cared for the patients. The preliminary results were presented at the 18th European AIDS Conference (EACS), 27–30 October 2021 (Poster AS-EACS-20221–00265).

Funding: the study was supported by the Gilead Fellowship Program 2017 (Principal Investigator: Prof. Giulia Marchetti), the grant ‘Ricerca Finalizzata 2019’ – Italian Ministry of Health (‘Significance and long-term clinical and virological evolution of cerebrospinal fluid HIV viral escape’, number RF-2019-12371089; PI: G. Marchetti) and by the grant ‘PSR2021_DIP_013’ of the Department of Health Sciences, University of Milan.

Author contribution: G.M., A.d.M., and F.B. developed the question research and the study protocol. F.M. helped with the study protocol writing and with the process of grant funding and Ethic Committee approval. V.B. designed and performed experiments. J.C. participated to laboratory experiments and interpretation. L.B. and F.B. performed neuropsychological evaluations. F.B. analyzed and interpreted the data and wrote the manuscript. C.F. and V.V. helped with patients’ recruitment and manuscript preparation. V.B., C.F., M.T., V.V., and A.C. helped in analyzing and interpreting the data. G.M., A.C., A.d.A., and E.V. contributed to the final data interpretation. All authors contributed to the editing of the manuscript. All authors read and approved the final manuscript.

### Conflicts of interest

There are no conflicts of interest.

## Supplementary Material

Supplemental Digital Content

## Supplementary Material

Supplemental Digital Content

## Supplementary Material

Supplemental Digital Content
