# Supplementary material for: Association between tight junction proteins and cognitive performance in untreated persons with HIV
Source: AIDS. 2024 May 2;38(9):1292–303. doi: 10.1097/QAD.0000000000003923 (PMC11216391; doi:10.1097/QAD.0000000000003923)
Supplement: Supplemental Digital Content [file aids-38-1292-s001.pdf]

Supplementary Figure 1. Inflammation and blood brain barrier impairment characterize patients with HAND

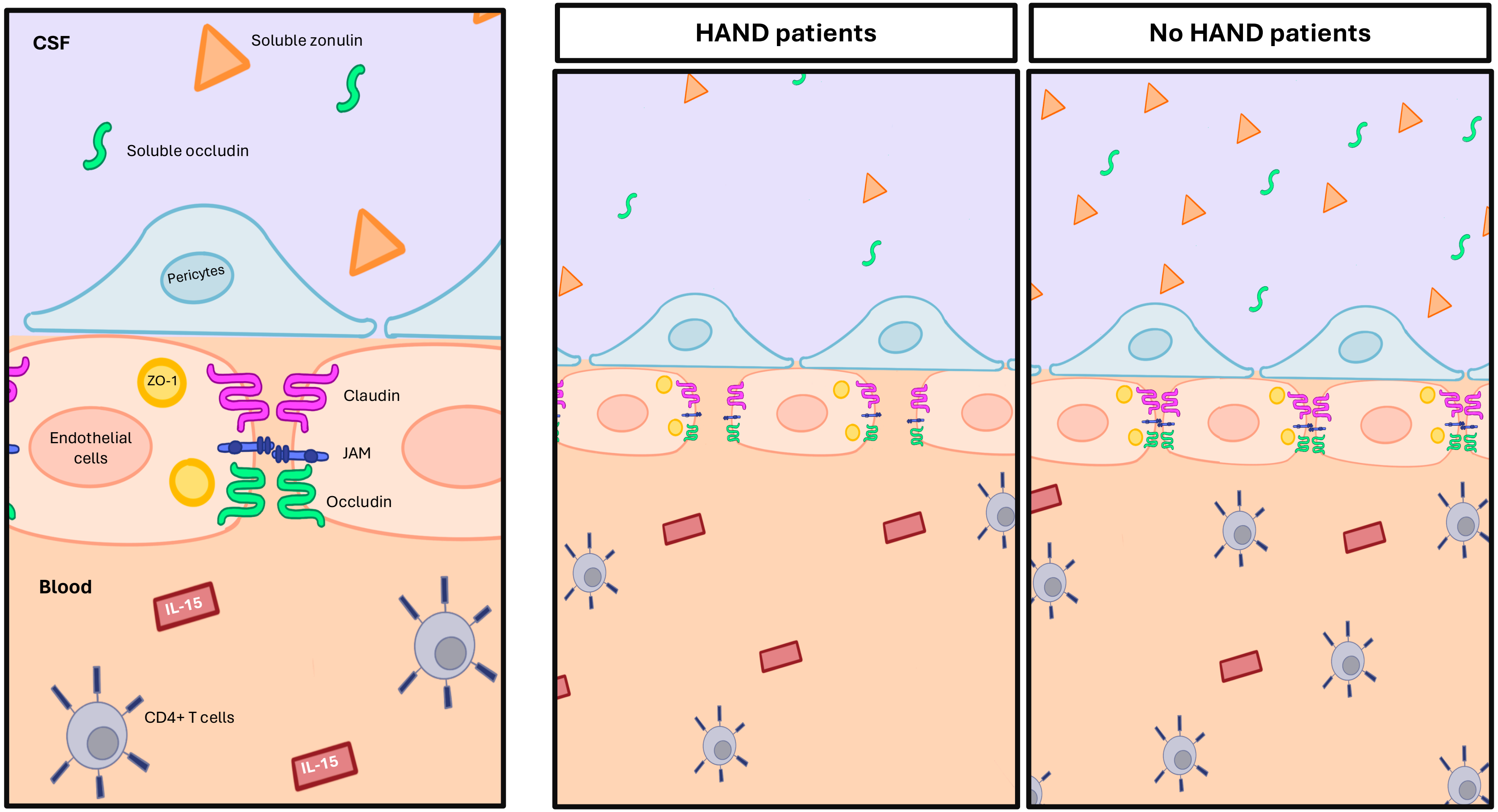

LEGEND: Inflammation and Blood Brain Barrier (BBB) impairment in patients with HIV-associated neurocognitive disorders (HAND) compared to patients without cognitive deficits. Exhaustion of CD4+ T cells, increased systemic inflammation together with a reduction of CSF zonulin and occludin levels feature patients with HAND.
