# Supplementary material for: Association between tight junction proteins and cognitive performance in untreated persons with HIV
Source: AIDS. 2024 May 2;38(9):1292–303. doi: 10.1097/QAD.0000000000003923 (PMC11216391; doi:10.1097/QAD.0000000000003923)
Supplement: Supplemental Digital Content [file aids-38-1292-s002.docx]

**Supplementary table 1 Results of the neurocognitive evaluation**

| ***Neurocognitive evaluation*** | **Study population**  **(N 78)** | |
| --- | --- | --- |
|  | **Raw scores^§^** | **T scores^§^** |
| *Attention and working memory* |  | 23,28 (5,88) |
| DST-F/B | 6,82 (1,9)/  5,97 (1,9) | 26,52 (9,92)/  26,24 (9,78) |
| CBTT | 6.23 (1,14) | 43,89 (10) |
| TMT-BA | 42.1 (28.2) | 0,88 (8,83) |
| *Speed of information processing* |  | 3,31 (3,05) |
| TMT-A | 30,89 (14,69) | 3,51 (9,94) |
| SCWT-T | 15,06 (11,45) | 3,97 (9,96) |
| SDMT | 48,19 (14,72) | 3,6 (9,81) |
| *Learning and memory* |  | 10,95 (8,17) |
| RAVLT-IR | 50,21 (9,9) | 5,05 (9,98) |
| RAVLT-DR | 9,79 (2,68) | 18,66 (9,99) |
| ROCF-DR | 18,23 (6,16) | 8,44 (9,98) |
| *Abstraction and executive functions* |  | 25,63 (4,96) |
| ROCF-C | 34,56 (2,49) | 20,36 (9,88) |
| TMT-B | 70,36 (34,68) | 1,32 (9,94) |
| SCWT-E | 0,62 (1,12) | 53,54 (1) |
| *Verbal fluency* |  | 4,15 (5,28) |
| SPFT-P/S | 37,39 (13,72)/  51,08 (13,5) | 3,87 (9,93)/  4,43 (1,01) |
| *Motor skills* |  | 6,13 (10,76) |
| FTT-R/L | 51,83 (7,88)/  48,72 (7,26) | 5,86 (11,57)/  6,41 (11,4) |

**Legend**

DST-F/B, Digit Span Test, Forward/Backward; CBTT, Corsi Block Tapping Task; TMT-A/-B/-BA, Trail Making Test, part A/part B/part BA; SCWT-T/-E, Stroop Color and Word Test, Time and Errors; SDMT, Symbol Digit Modalities Test; RAVLT-IR/DR, Rey Auditory Verbal Learning Test, Immediate Recall and Delayed Recall; ROCF-DR/C, Rey-Osterrieth Complex Figure test, Delayed Recall and Copy; SPFT-P/S, Semantic and Phonemic Fluency Task, Phonemic/Semantic; FTT-R/L, Finger Tapping Test, Right/Left.

The raw scores obtained at the neuropsychological tests are corrected for age, educational level and gender in the Italian population and then converted to normative T scores. Cognitive domain T-scores were calculated averaging the T-scores of the single tests.

^§^Data are presented as mean (standard deviation).
