# Supplementary material for: Association between tight junction proteins and cognitive performance in untreated persons with HIV
Source: AIDS. 2024 May 2;38(9):1292–303. doi: 10.1097/QAD.0000000000003923 (PMC11216391; doi:10.1097/QAD.0000000000003923)
Supplement: Supplemental Digital Content [file aids-38-1292-s003.docx]

**Supplementary Table 2 Association between plasma and CSF biomarkers and the mean T-scores of the neurocognitive evaluation by fitting linear regression analyses**

| **Parameters** |  |  |  |  |  |  |
| --- | --- | --- | --- | --- | --- | --- |
| 1. **Attention and working memory** | **β Coefficient** | **CI 95%** | **p values** | **β Coefficient** | **CI 95%** | **p values** |
|  | **Plasmatic levels** | | | **CSF levels** | | |
| IL-15, log_10_ pg/mL | -0,110 | -4,389; 1,829 | 0,413 | -0,373  -0,404* | -11,548; -1,796  -12,821; -1,540* | **0,008**  **0,014*** |
| TNF-α, log_10_ pg/mL | 0,208 | -0,887; 3,460 | 0,237 | -0,078 | -1,132; 0,740 | 0,672 |
| VCAM-1, log_10_ ng/mL | -0,088 | -1,281; 0,647 | 0,512 | -0,145 | -10,113; 2,953 | 0,277 |
| ICAM-1, log_10_ ng/mL | 0,015 | -6,924; 7,724 | 0,913 | -0,015 | -6,872; 6,235 | 0,922 |
| Claudin-5, log_10_ ng/mL | -0,054 | -1,792; 1,194 | 0,690 | 0,083 | -0,782; 1,489 | 0,535 |
| Occludin, log_10_ ng/mL | -0,009 | -17,220; 16,359 | 0,959 | -0,105 | -18,39; 10,067 | 0,556 |
| Zonulin, log_10_ ng/mL | 0,140 | -1,057; 2.,23 | 0,430 | -0,199 | -14,611; 4,076 | 0,259 |
| **b)** **Speed of information processing** | **β Coefficient** | **CI 95%** | **p values** | **β Coefficient** | **CI 95%** | **p values** |
|  | **Plasmatic levels** | | | **CSF levels** | | |
| IL-15, log_10_ pg/mL | -0,004 | -1.668; 1,621 | 0,977 | -0,044 | -3,277; 2,440 | 0,769 |
| TNF-α, log_10_ pg/mL | -0,461  -0,281* | -2,950; -0,510  -9,250; 2,124* | **0,007**  0,207* | -0,001 | -0,581; 0,579 | 0,997 |
| VCAM-1, log_10_ ng/mL | -0,117 | -0,757; 0,300 | 0,390 | -0,335  -0,336* | -7,488; -0,992  -7,259; 0,431* | **0,011**  0,080* |
| ICAM-1, log_10_ ng/mL | 0,033 | -3,349; 4,274 | 0,809 | -0,503  -0,436* | -7,228; -2,149  -6,547; -0,526* | **<0,001**  **0,023*** |
| Claudin-5, log_10_ ng/mL | -0,382  -0,552* | -1,810; -0,371  -1,727; -0,637* | **0,004**  **<0,001*** | -0,047 | -0,701; 0,493 | 0,728 |
| Occludin, log_10_ ng/mL | -0,06 | -12.087; 8,657 | 0,738 | -0,483  -0,271* | -19,646; -3,962  -16,878; 4,332* | **0,004**  0,232* |
| Zonulin, log_10_ ng/mL | 0,125 | -0,706; 1,447 | 0,488 | -0,232 | -9,443; 2,005 | 0,195 |
| **c) Learning and memory** | **β Coefficient** | **CI 95%** | **p values** | **β Coefficient** | **CI 95%** | **p values** |
|  | **Plasmatic levels** | | | **CSF levels** | | |
| IL-15, log_10_ pg/mL | -0,167 | -6,954; 1,209 | 0,165 | -0,289  -0,148 | -14,791;-0,942  -9,665; 2,673 | **0,027**  0,260 |
| TNF-α, log_10_ pg/mL | 0,165 | -1,758; 5,212 | 0,322 | -0,184 | -2,054; 0,620 | 0,284 |
| VCAM-1, log_10_ ng/mL | -0,251  0,058* | -2,261; -0,085  -1,021; 1,583* | **0,035**  0,667* | -0,066 | -10,524; 5,958 | 0,582 |
| ICAM-1, log_10_ ng/mL | -0,035 | -10,236; 7,236 | 0,772 | -0,029 | -7,996; 6,494 | 0,836 |
| Claudin-5, log_10_ ng/mL | 0,191 | -0,385; 3,650 | 0,111 | -0,048 | -1,858; 1,242 | 0,693 |
| Occludin, log_10_ ng/mL | 0,043 | -26,123; 33,655 | 0,800 | 0,330  0,120* | 0,727; 43,778  -14,983; 33,446* | **0,043**  0,440* |
| Zonulin, log_10_ ng/mL | 0,146 | -1,560; 3,977 | 0,382 | 0,264 | -2,791; 26,542 | 0,109 |
| **d) Abstraction and executive functions** | **β Coefficient** | **CI 95%** | **p values** | **β Coefficient** | **CI 95%** | **p values** |
|  | **Plasmatic levels** | | | **CSF levels** | | |
| IL-15, log_10_ pg/mL | 0,141 | -1,223; 3,995 | 0,292 | 0,197 | -1,540; 26,676 | 0,175 |
| TNF-α, log_10_ pg/mL | -0,640  -0,137* | -5,559; -2,206  -13,145; 7,054* | **<0,001**  0,537* | -0,264 | -1,515; 0,233 | 0,145 |
| VCAM-1, log_10_ ng/mL | -0,016 | -0,864; 0,765 | 0,903 | -0,164 | -8,905; 2,086 | 0,219 |
| ICAM-1, log_10_ ng/mL | 0,070 | -4,536; 7,772 | 0,600 | -0,120 | -5,258; 2,291 | 0,432 |
| Claudin-5, log_10_ ng/mL | -0,109 | -1,764; 0,741 | 0,417 | -0,074 | -1,225; 0,695 | 0,582 |
| Occludin, log_10_ ng/mL | 0,066 | -0,711; 19,477 | 0,711 | -0,159 | -20,07; 7.679 | 0,370 |
| Zonulin, log_10_ ng/mL | 0,0001 | -1,728; 1,724 | 0,998 | 0,061 | -7,768; 10,926 | 0,733 |
| **e) Verbal fluency** | **β Coefficient** | **CI 95%** | **p values** | **β Coefficient** | **CI 95%** | **p values** |
|  | **Plasmatic levels** | | | **CSF levels** | | |
| IL-15, log_10_ pg/mL | -0,234  -0,211* | -5,224; -0,022  -4,855; 0,437* | **0,048**  0,100 | -0,119 | -6,684; 2,490 | 0,364 |
| TNF-α, log_10_ pg/mL | 0,227 | -0,649; 3,656 | 0,165 | -0,343  -0,270* | -1,573; -0,050  -1,422; 0,300* | **0,037**  0,191* |
| VCAM-1, log_10_ ng/mL | 0,016 | -0,676; 0,775 | 0,891 | -0,038 | -6,180; 4,478 | 0,751 |
| ICAM-1, log_10_ ng/mL | -0,080 | -7,649; 3,804 | 0,505 | 0,036 | -4,115; 5,351 | 0,794 |
| Claudin-5, log_10_ ng/mL | 0,138 | -0,544; 2,081 | 0,247 | 0,005 | -0,826; 0,864 | 0,964 |
| Occludin, log_10_ ng/mL | 0.157 | -8,274; 23,438 | 0,339 | 0,370  0,308* | 2,593; 29,019  -2,774; 34,294* | **0,020**  0,092* |
| Zonulin, log_10_ ng/mL | -0,067 | -2,098; 1,394 | 0,685 | 0,246 | -2,202; 16,208 | 0,132 |
| **f) Motor skills** | **β Coefficient** | **CI 95%** | **p values** | **β Coefficient** | **CI 95%** | **p values** |
|  | **Plasmatic levels** | | | **CSF levels** | | |
| IL-15, log_10_ pg/mL | -0,073 | -7,098; 3,779 | 0,545 | -0,295  -0,232* | -19,950; -1,586  -15,957; 0,105* | **0,022**  0,053* |
| TNF-α, log_10_ pg/mL | -0,065 | -5,447; 3,654 | 0,692 | -0,376  -0,127* | -3,540; -0,298  -1,998; 0,811* | **0,022**  0,392* |
| VCAM-1, log_10_ ng/mL | -0,471  -0,378* | -4,230; -1,621  -4,190; -0,886* | **<0,001**  **0,003*** | -0,335  -0,322* | -25,533; -5,050  -25,636; -2,394* | **0,004**  **0,019*** |
| ICAM-1, log_10_ ng/mL | 0,019 | -10,762; 12,654 | 0,872 | -0,286  -0,304* | -20,701; -0,827  -21,770; -1,205* | **0,034**  **0,029*** |
| Claudin-5, log_10_ ng/mL | -0,003 | -2,740; 2,662 | 0,977 | -0,012 | -1,810; 1,635 | 0,920 |
| Occludin, log_10_ ng/mL | 0,225 | -9,880; 54,685 | 0,168 | -0,272 | -52,206; 4,287 | 0,094 |
| Zonulin, log_10_ ng/mL | 0,179 | -1,612; 5,495 | 0,275 | 0,036 | -17,493; 21,675 | 0,830 |

**LEGEND**

Association between plasma and CSF biomarkers (inflammation, endothelial adhesion molecules and Blood Brain Barrier impairment) and neurocognitive performance by fitting a linear regression analysis.

The raw scores obtained at the neuropsychological tests are corrected for age, educational level and gender in the Italian population and then converted to normative T scores. Cognitive domain T-scores were calculated averaging the T-scores of the single tests: **attention and working memory**: DST-F/B, Digit Span Test, Forward/Backward; CBTT, Corsi Block Tapping Task; TMT-BA, Trail Making Test, part BA - **speed of information processing**: TMT-A, Trail Making Test, part A; SCWT-T, Stroop Color and Word Test, Time; SDMT, Symbol Digit Modalities Test – **learning and memory**: RAVLT-IR/DR, Rey Auditory Verbal Learning Test, Immediate Recall and Delayed Recall; ROCF-DR, Rey-Osterrieth Complex Figure test, Delayed Recall – **abstraction and executive functions**: ROCF-DR/C, Rey-Osterrieth Complex Figure test, Copy; TMT-B, Trail Making Test, part B; SCWT-T/-E, Stroop Color and Word Test, Errors – **verbal fluency**: SPFT-P/S, Semantic and Phonemic Fluency Task, Phonemic/Semantic; **motor skills**: FTT-R/L, Finger Tapping Test, Right/Left.

CI95%, 95% confidence interval. CSF: cerebrospinal fluid.

*Multivariable linear regression analysis, adjusted for age, CD4+ T cells nadir, CSF/plasma HIV-RNA ratio and AIDS events.
